# Visualizing heterogeneous associations: economic capital and adolescent football participation in China: a cross-sectional study using quantile regression with school-level fixed effects

**DOI:** 10.3389/fpsyg.2026.1798818

**Published:** 2026-05-29

**Authors:** Donghui Jin, Zhengri Quan, Dan Pang, Nanhu Li

**Affiliations:** 1 School of Physical Education, Kookmin University, Seoul, Republic of Korea; 2 School of Physical Education and Health, Changchun Normal University, Changchun, China; 3 School of Sports Science, China Three George University, Yichang, China

**Keywords:** adolescent health, fixed effects model, health equity, quantile regression, socioeconomic factors, sport participation

## Abstract

**Background:**

Fair participation in sports is a key focus of public health, but socioeconomic barriers limit youth participation. Although family wealth is related to participation, its specific differences at different levels of opportunity are not yet clear. This study goes beyond the average correlation and deeply analyzes the differentiated relationship between adolescent family economic capital and opportunities for football participation.

**Methods:**

We conducted a cross-sectional survey among 1,860 adolescents aged 12–18 years (from 42 schools) in Jilin Province. The sample was selected by multi-stage stratified cluster sampling. Data were collected using a standardized questionnaire. The study constructs a comprehensive “Football Participation Opportunity Index” and “Family Economic Capital Index.” After controlling for a range of personal, family, and environmental factors, we estimated the coefficients of economic capital at the 10th, 25th, 50th, 75th, and 90th quantiles of the FOI distribution using quantile regression models with school fixed effects. At the same time, we have drawn a series of charts to visualize the data base, methodological principles, core findings, potential mechanisms and policy implications.

**Results:**

The analysis revealed a significant J-type association pattern. The impact of economic capital was strongest in the group with the lowest chance of participation (10th quantile; β = 0.341, 95% CI: 0.250–0.432), suggesting that it may constitute a critical participation threshold. The effect weakened around the median but strengthened again at the top of the chance distribution (90th quantile; β = 0.260, 95% CI: 0.145–0.375), suggesting that it may be related to access to elite-level opportunities. Visualization analysis further illustrates that other factors, such as urban household registration, parental contacts and school facilities, also show different patterns of association throughout the opportunity spectrum.

**Conclusion:**

The correlation between family economic capital and youth football participation shows a significant random level change, revealing inequality at both ends. We need to adopt a dual track strategy: providing economic support for disadvantaged youth, while ensuring transparency and merit based selection in elite selection. The visualization framework enhances the understanding and conversion potential of discoveries.

## 1 Introduction

Promoting regular physical activity among adolescents is a cornerstone of global public health efforts because of its clear benefits for cardiometabolic health, mental health, and social development (Poitras et al., 2016; Guthold et al., 2020). Football, as a global popular team sport, is an important way for young people to achieve the recommended level of sports and obtain related health benefits (Eather et al., 2023). However, opportunities to participate in organized sport are not equitably distributed and often reflect broader socio-economic differences. Socioeconomic status has been consistently identified as a factor associated with sport participation, with higher participation rates among children from more affluent families (Owen et al., 2022; Gautam et al., 2023). This problem is particularly prominent in the context of contemporary China. In the specific context of youth football in China, participation opportunities are typically organized through two main channels: state-funded sports programs within public schools, and extracurricular activities that may include school teams, community clubs, or private academies. While the former is usually free of charge, participation in the latter, particularly in private or competitive training settings, usually requires parental payment (Chinese Football Association, 2016). The government has launched ambitious national strategies, such as the “Football Reform and Development” program and the inclusion of sports in the high school entrance examination, explicitly aimed at fostering sports culture and improving the physical fitness of young people (Wu et al., 2024). However, profound socioeconomic disparities, including the urban-rural divide and significant regional development gaps, pose a major challenge to the equitable achievement of these goals (Dong et al., 2019; Jiang et al., 2023). In this unique socio-political landscape, the role of the family in securing developmental opportunities for its children—often referred to as “collaborative parenting”—has become increasingly prominent (Miranda, 2006). Family economic capital may translate into competitive advantages (e.g., access to personal trainers, elite football academies, and expensive qualification pathways), which may inadvertently create new forms of inequality that may undermine the inclusive spirit of public health policies (Zheng et al., 2025).

Although the general association between SES and sport participation is well documented (Andersen et al., 2022; Harris et al., 2024), the dominant analytical approach in the literature relies on mean-based regression models such as ordinary least squares (OLS). This approach paints an incomplete picture by estimating average associations that may mask critical heterogeneity across the population. It fails to answer a central question that is particularly critical for a society in transition like China: Is the relationship between economic resources and football participation consistent, or is there a fundamental difference in its intensity and nature between adolescents at the bottom and those at the top of the distribution of opportunities? The concept of “economic capital” is thought to be associated with multifaceted barriers (Pérez-Stable and Webb Hooper, 2023), but its potentially non-unifying role—a barrier for some, a ladder for others—is still not adequately captured by traditional approaches that focus only on average effects. Quantile regression fills this critical gap by modeling relationships at specific points (quantiles) of the conditional distribution of outcomes (Peng, 2021). This approach is particularly suited to studying inequality because it can reveal whether factors such as economic capital act most powerfully as “barriers to entry” at the low end of the opportunity spectrum or as “escalators” at the high end—a key distinction that average-based models such as OLS fail to detect. However, the presentation of quantile regression results often relies on coefficient tables, which may not adequately convey the subtlety, continuity, and underlying mechanisms of heterogeneous associations (Khadka et al., 2024). Effective data visualization is therefore essential for deciphering complex patterns, communicating scientific findings, and translating them into actionable insights.

To this end, this study uses a representative sample from Jilin Province in Northeast China (where sports culture is strong, but socio-economic diversity is also significant) to go beyond the average correlation and deeply analyze the complex relationship of economic capital. We use an integrated approach to analysis and visual storytelling. First, we use quantile regression combined with school-level fixed effects to test a central hypothesis: that economic capital is not uniformly associated with football opportunities, but instead shows the strongest association for both youth at the bottom of the opportunity distribution (suggesting a threshold effect) and youth at the top (suggesting a promotion effect). Second, we generate a series of visual diagrams tailored to: (1) clarify the need for our methodological choices; (2) dramatize the finding of heterogeneous associations across the entire opportunity spectrum; (3) propose a conceptual framework of dual-path mechanisms that may connect economic capital to different outcomes; (4) map different patterns of association of key covariates; and (5) map the relationship between the two outcomes; And (5) explain the potential comparative significance of different policy approaches based on our findings. The integrated findings aim to provide a nuanced and visually understandable evidence base to inform the design of targeted public health interventions to address the different manifestations of inequality in sport in China and similar contexts.

## 2 Materials and methods

### 2.1 Study design and population

This cross-sectional study is part of the Youth Health and Physical Education Development Survey 2024. The subjects were adolescents aged 12–18 years old who were studying in public schools in Jilin Province. The sample size calculation is targeted to ensure 90% test power (α = 0.05) and takes into account the complex survey design and the planned modeling strategy (Moyé, 2016). Participants were excluded if they had medical contraindications to physical activity or provided incomplete responses to key survey variables.

### 2.2 Sampling strategy

A multi-stage stratified cluster random sampling method was used to ensure the representativeness of the sample at the provincial level (Flege and Thomsen, 2018). In the first stage, all counties and districts in Jilin Province are divided into three different levels according to the level of urbanization: urban, suburban and rural. In the second stage, a certain number of junior high schools and senior high schools were randomly selected from each level as primary sampling units (clusters). In the final stage, one or two complete classes were randomly selected from each grade in each selected school, and all students in these classes were invited to participate in the survey. The process resulted in a sample of 1,860 students from 42 schools, with a response rate of 92.5%. All participants and their guardians signed an informed consent form. Ethical approval of the study protocol was obtained from the Institutional Review Board of Changchun Normal University.

### 2.3 Data collection and measurement

Data collection was accomplished through a self-administered structured electronic questionnaire, which was distributed during the prescribed school program hours and supervised by trained researchers on site to ensure data quality and standardization of procedures (Mozersky et al., 2020).

#### 2.3.1 Dependent variable: football participation opportunity index (FOI)

FOI is a multi-dimensional comprehensive index constructed by principal component analysis to measure the breadth, depth and quality of participation (Jolliffe and Cadima, 2016). It integrates three specific indicators:

*Breadth:* a binary indicator of involvement in any organized football activity (training or playing) within the last 12 months. This encompasses participation both within the standard school physical education curriculum and in extracurricular settings, which may include school teams, community clubs, or private academies where parental fees are often required.
*Depth:* The average number of hours per week devoted to football-specific training or competition.
*Quality:* a binary indicator of access to licensed football coaches.

The first principal component, which explained 68% of the total variance, was retained as a continuous FOI score. To assess the validity of this composite index, we examined the correlation between it and the global self-assessment of individual football opportunities (Spearman’s Rho = 0.71, *p* < 0.001), providing evidence of convergent validity. In addition, the selection of indicators refers to the relevant review of youth sports participation framework to support content validity (Jolliffe and Cadima, 2016). While this approach captures multidimensional information, we acknowledge the potential challenges of its direct interpretation and conducted a sensitivity analysis using alternative outcome definitions to ensure robustness. To provide evidence of criterion validity, we examined the association of FOI with participants’ overall physical activity levels as measured by the short version of the IPAQ-S (Tran et al., 2020). As expected, FOI was significantly and positively correlated with total metabolic equivalent minutes per week (Spearman’s ρ = 0.45, *p* < 0.001), supporting its validity as a measure of sports participation.

#### 2.3.2 Core independent variable: family economic capital index (ECI)

ECI is similarly constructed using PCA to create a robust measure that goes beyond mere revenue (Filmer and Pritchett, 2001). It combines three self-reported components:

*Family Possessions:* a standardized count of the household’s high-value assets (e.g., family car, personal computer).
*Parental education:* The highest level of education attained by either parent (ordered scale).
*Subjective family affluence:* a single measure of perceived family economic status relative to peers.

The first principal component in this analysis, which explained 72% of the variance, was used as the continuous ECI score. This asset-based index method is widely used in the context of poor reliability of income data. The internal consistency of the three components was acceptable (Cronbach’s alpha = 0.68).

#### 2.3.3 Covariates

The study included a comprehensive set of covariates to control for potential confounders at multiple levels: *Individual characteristics:* age, gender, domicile (urban/rural), only child or not, self-rated health status (5-point Likert scale) (Currie et al., 2023), and BMI category based on Chinese standards (Zhang et al., 2018).

*Family capital factors:* parents’ education level (junior college and above vs. below), parents’ sports participation, the number of family sports equipment, parents’ social network in sports organizations.
*School and environment factors:* perceived adequacy of school sports facilities, peer support for sports activities, weekly academic stress (all 5-point scales), and accessibility of community sports centers. These school-level factors were then used to construct a fixed-effects model.

### 2.4 Statistical analysis and visualization

**FIGURE 1:**
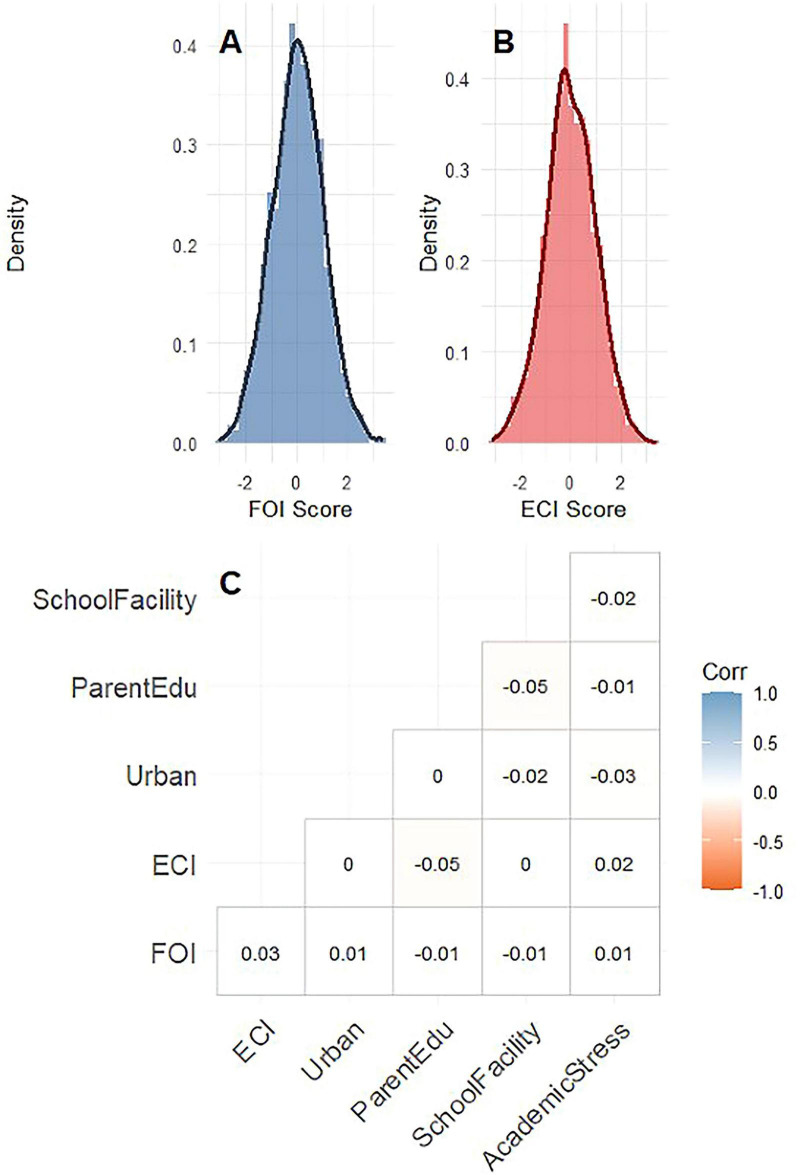
Distribution and correlation of key variables. **(A)** Histogram of the distribution of the Football Participation Opportunity Index (FOI) for the total sample (*n* = 1,860) with the density curve superimposed. **(B)** Family of the Economic Capital Index (ECI) distribution with the density curve superimposed. **(C)** Thermal plot of the correlation matrix showing the Spearman correlation coefficient between FOI, ECI and key covariates (city residence, parental education, adequacy of school facilities, academic stress) (lower triangle). The color shade and size of the dots correspond to the size of the correlation coefficient. The data showed an approximately normal distribution, and a correlation matrix revealed a preliminary bivariate association, indicating that higher FOI was positively associated with urban residence, higher parental education, and better school facilities, and negatively associated with academic stress.

The analysis began with descriptive statistics, which were calculated for the total sample and stratified by the tertiles of the Football Participation Opportunity Index. For continuous variables, analysis of variance was used to test for differences between stratified groups; for categorical variables, the chi-square test was used. To visualize the distribution of key variables and their pairwise relationships, we generated Figure 1 using the ggplot2 package of the R software. The figure includes: (A) a histogram of the football participation opportunity index distribution with density coverage; (B) a histogram of the household economic capital index distribution with density coverage; and (C) a thermodynamic map (generated using the ggcorrplot package) demonstrating the Spearman correlation coefficients between the football participation opportunity index, the household economic capital index, and key covariates.

**FIGURE 2:**
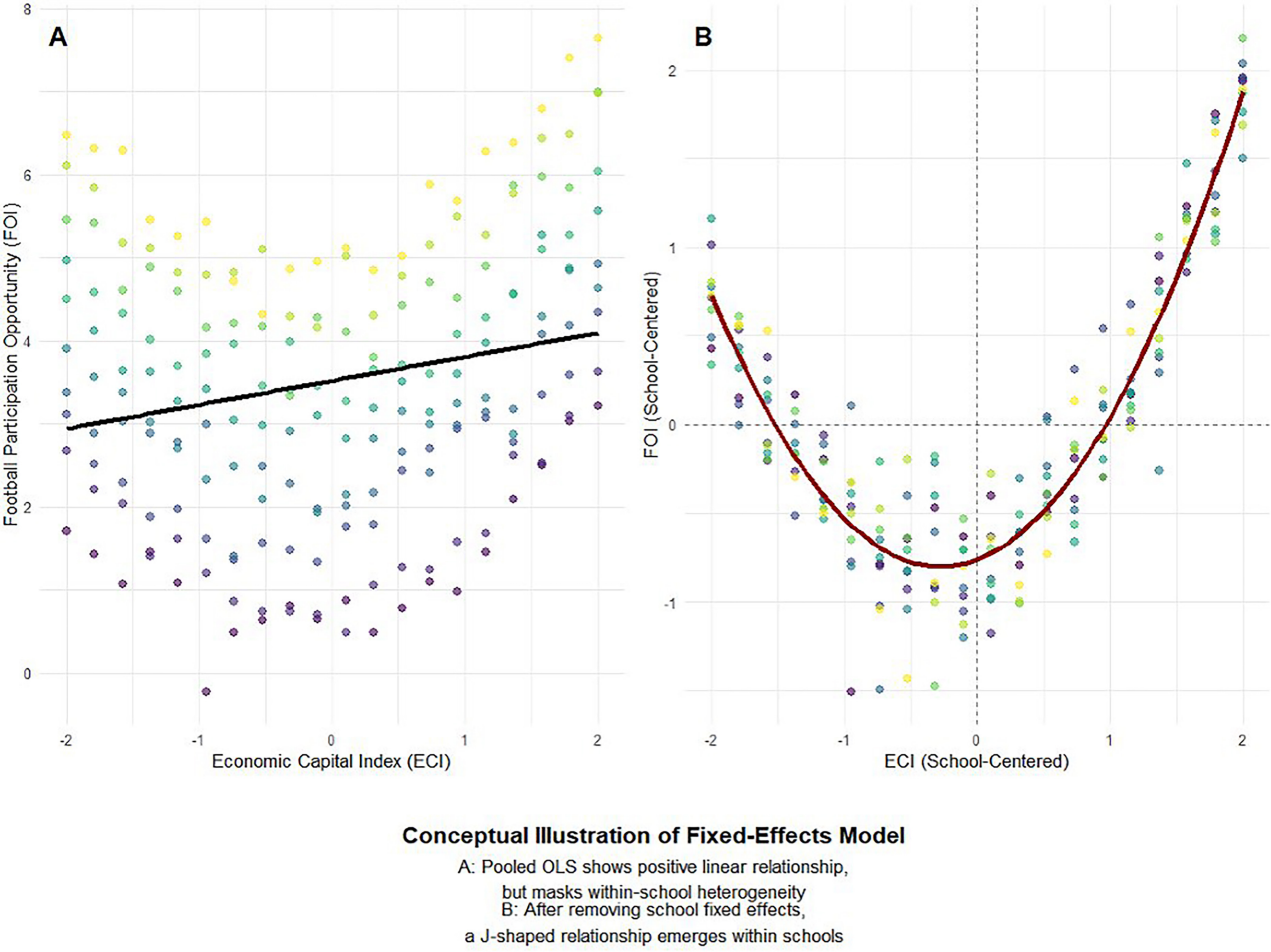
Conceptual illustration of fixed-effects model necessity. **(A)** A mixed ordinary least squares (OLS) regression model showing the relationship of the economic capital index to football participation opportunities across all schools. Each color represents a different school. The black linear fit line illustrates the “average effect” but masks significant inter-school heterogeneity. **(B)** Intramural relationships after applying school-level fixed effects. By centralizing ECI and FOI around the mean of their respective schools, a clear J-type quadratic relationship (red curve) emerges after removing the school-specific factors that do not change over time. This visualization shows how traditional OLS may mask significant intramural heterogeneity, thereby demonstrating the need to use fixed-effects quantile regression to capture contextual dependencies.

Central to the analysis was the application of a quantile regression model incorporating school-level fixed effects, which was achieved by including a set of school dummy variables. This approach controls for unobserved heterogeneity (e.g., school culture, district sports policy environment) that does not change over time at the school level by comparing students within the same school. The model is: Q _ τ (FOI _ IJ | X) = β _ 0τ + β _ 1τ (ECI _ IJ) + Σβ _ kτ (Covariates _ IJ) + Σθ _ J (School Dummy _ J). Where Q _ τ represents the τ-th conditional quantile of the football participation opportunity index for student “I” in school “J”; beta _ 1τ is the coefficient of our primary interest in the quantile τ; and X is the vector of all covariates. To conceptually illustrate the need for this approach, we create Figure 2. The figure contrasts the pooled OLS regression (Figure 2A) with the intra-school relationships revealed after applying fixed effects (Figure 2B) using simulated data, clearly demonstrating the heterogeneity that is masked. We estimated this model for τ = (0.10, 0.25, 0.50, 0.75, 0.90). Standard errors were estimated using the bootstrap method, which was repeated 500 times to ensure its reliability (Lu and Fan, 2020). The Wald test was used to formally test the equality of the coefficients across different quantiles. We acknowledge that testing for multiple quantiles and covariates increases the risk of making Type I errors; the findings should be read as exploratory patterns.

**FIGURE 3:**
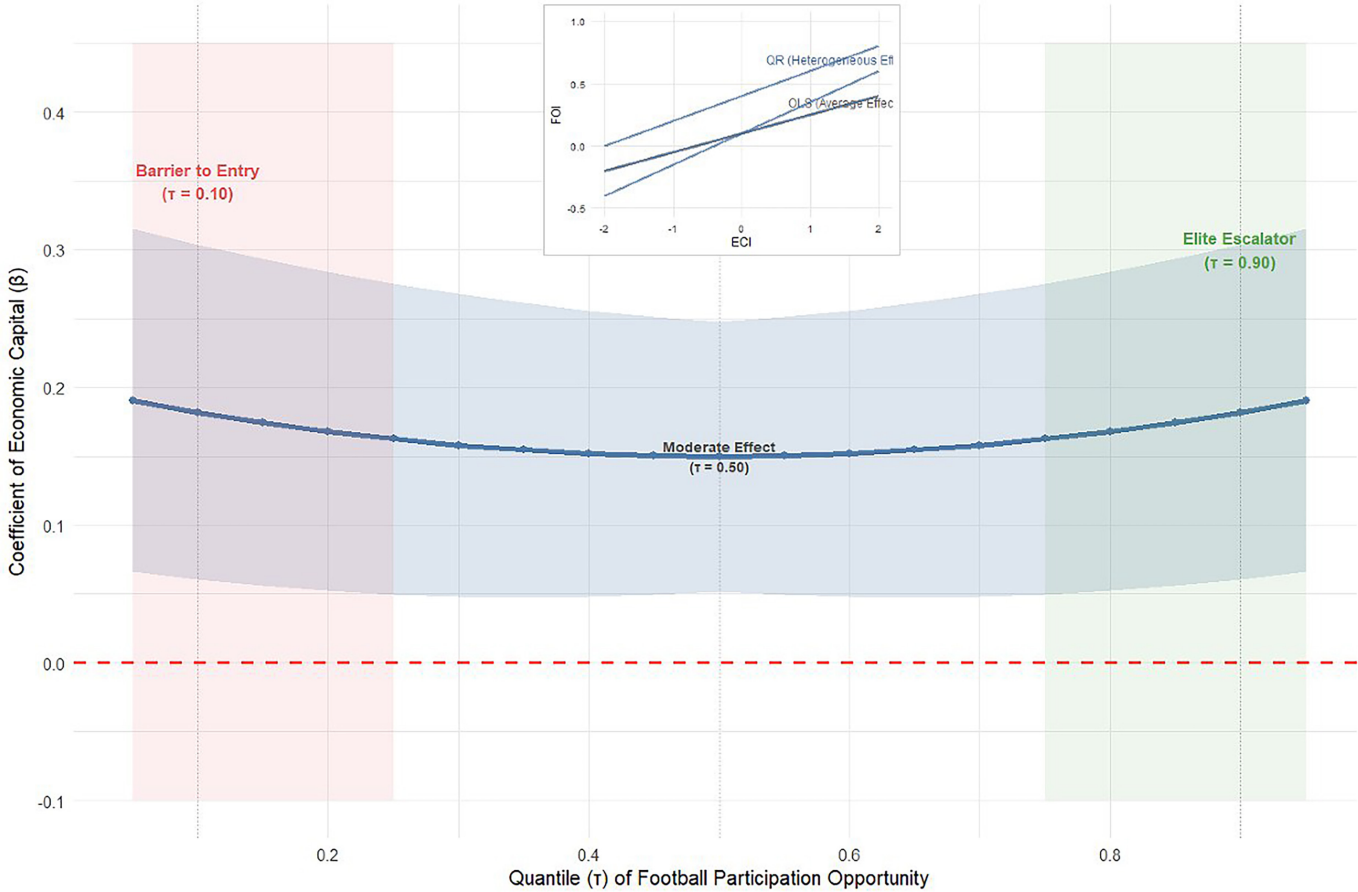
Heterogeneous effects of economic capital on football participation across quantiles (J-shaped pattern). This figure shows the economic capital index coefficient (β) from the fixed-effects quantile regression model over the conditional distribution of the football participation opportunity index (quantile τ from 0.05 to 0.95). The solid blue line represents the point estimate and the shaded area indicates the 95% confidence interval. The red dashed line at y = 0 indicates no effect. The apparent J-shaped pattern reveals that economic capital exerts its strongest influence at both ends of the opportunity distribution: as a “barrier to entry” at the lowest quantile (τ = 0.10) and as an “elite promotion ladder” at the highest quantile (τ = 0.90), with its effect weakening around the median (τ = 0.50). The embedded plot in the upper left corner contrasts the single slope (mean effect) estimated by OLS with the multiple change slopes captured by quantile regression, illustrating the methodological advantage of capturing heterogeneous effects.

The main result of this analysis, the J-shaped pattern of the economic capital coefficient as a function of quantile, is presented in Figure 3. The figure plots the point estimates and their 95% confidence intervals for the coefficients of household economic capital in quantile regression models with school fixed effects over the range τ = 0.05–0.95. The illustration is enhanced by the labeling of the key quantiles (τ = 0.10, 0.50, 0.90) and the hatching of the “Barriers to Access” and “Elite Ladder” regions. A vignette was also added to visually contrast the single slope of the OLS with the multiple slopes of the quantile regression.

**FIGURE 4:**
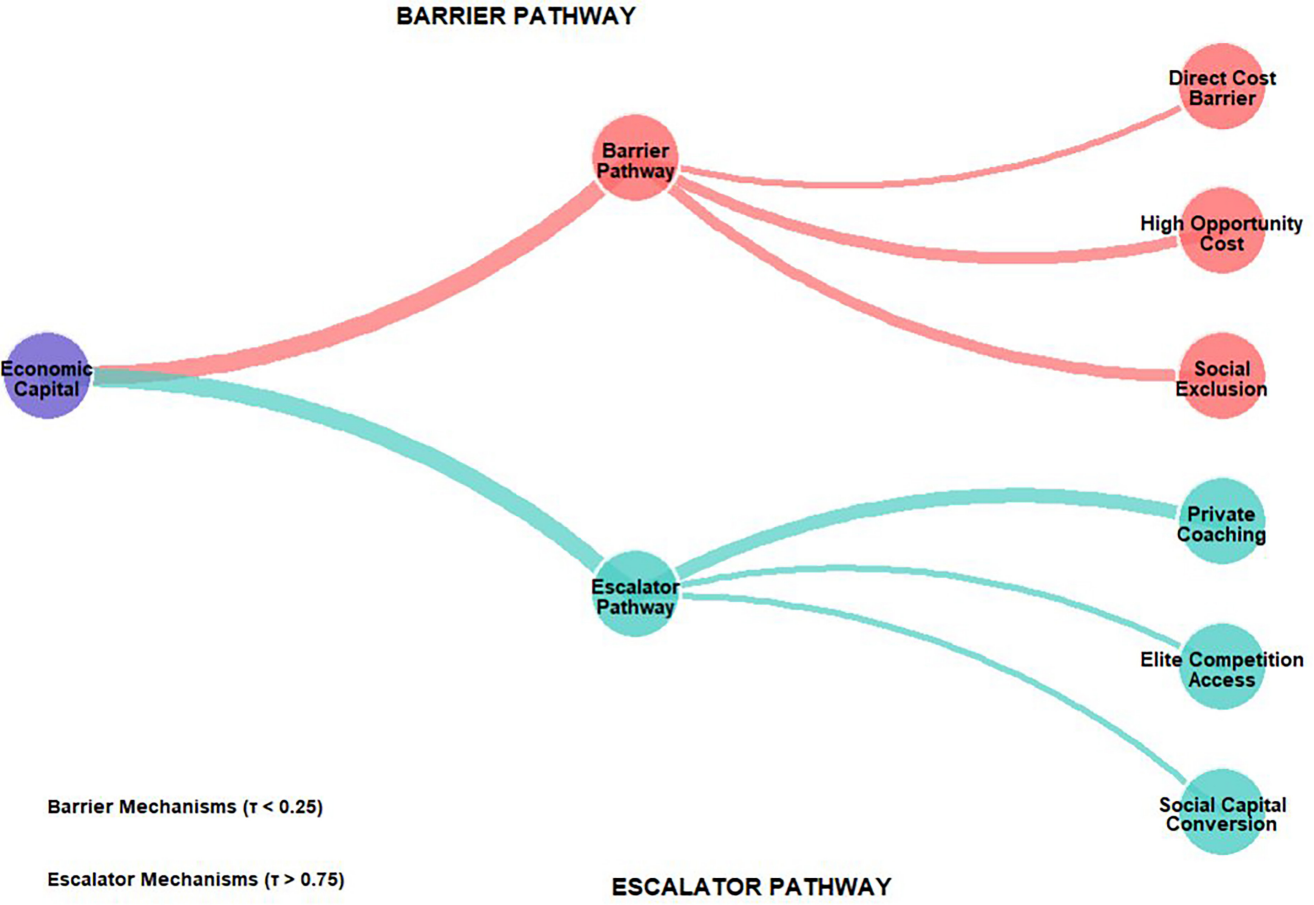
Mechanisms of economic capital’s heterogeneous effects (dual pathways: barrier vs. escalator). This diagram conceptualizes the dual pathways by which family economic capital differentiation affects football participation opportunities. The central source (“economic capital”) bifurcates into two distinct paths: (1) the “barrier path” on the left (red) illustrates the mechanism that affects adolescents at the bottom of the opportunity distribution (τ < 0.25), Economic deprivation contributes to “direct cost barriers” (equipment/expenses), “high opportunity costs” in academically prioritized cultures, and perceptions of “social exclusion.” (2) The “promotion ladder path” (blue) on the right side illustrates the mechanism that affects adolescents at the top of the distribution (τ > 0.75), where economic surplus enables “private coach guidance,” “elite competition access” and “social capital transformation.” The curve connections show the scaling between the phases, and the path width indicates the relative strength. This framework goes beyond statistical correlation and proposes testable mechanisms to explain J-shaped patterns.

In order to propose a theoretical framework to explain the J-pattern, we constructed a conceptual mechanism map (Figure 4). This schematic, created using ggplot2 geometric objects, illustrates the dual path hypothesis that economic capital may be associated with different outcomes: for low-opportunity adolescents, it acts as an “access barrier” (through direct costs, opportunity costs, and social exclusion). For high-opportunity adolescents, it serves as an “elite promotion ladder” (through personal coaches, elite channel access and social capital transformation). This framework is presented as a proposition for future longitudinal or qualitative studies to validate.

**FIGURE 5:**
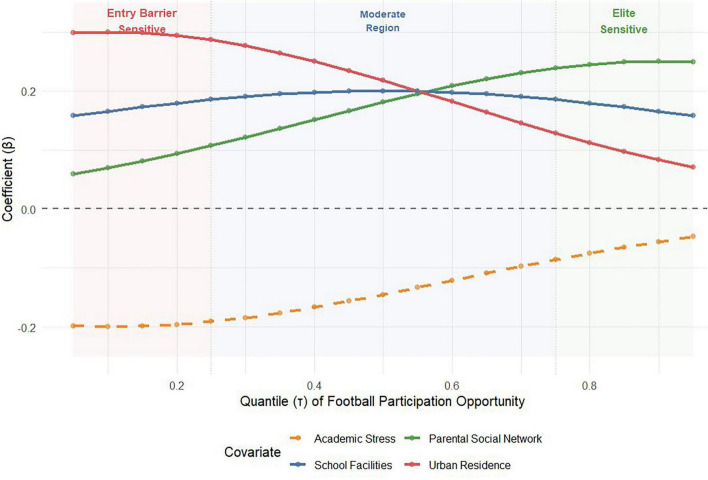
Heterogeneous effects of key covariates across the opportunity spectrum. This figure shows the trajectory of the regression coefficient (β) for four representative covariates from the fixed-effects quantile regression model across the conditional distribution of football participation opportunity (quantile τ from 0.05 to 0.95). The analysis revealed three different types of effects: (1) The “access-sensitive” factors (urban residence and academic stress) showed the strongest effect at lower quantiles (τ < 0.25) and weakened at higher quantiles. (2) The “elite-sensitive” factor (parental social network) is significantly enhanced at higher quantiles (τ > 0.75). (3) The “universal facilitative” factor (school facilities) maintained a consistent positive association across all quantiles. The background shaded area divides the three intervals of the chance distribution, and the corresponding labels highlight the areas where each factor plays a major role.

To examine the heterogeneous association of covariates, we extended quantile regression with fixed effects to all covariates and plotted the trajectory of the coefficients for four representative variables over the entire quantile range (τ = 0.05–0.95). Figure 5 demonstrates these trajectories, allowing for a visual classification of covariates into categories such as “access-sensitive,” “elite-sensitive,” and “universally-facilitated.”

**FIGURE 6:**
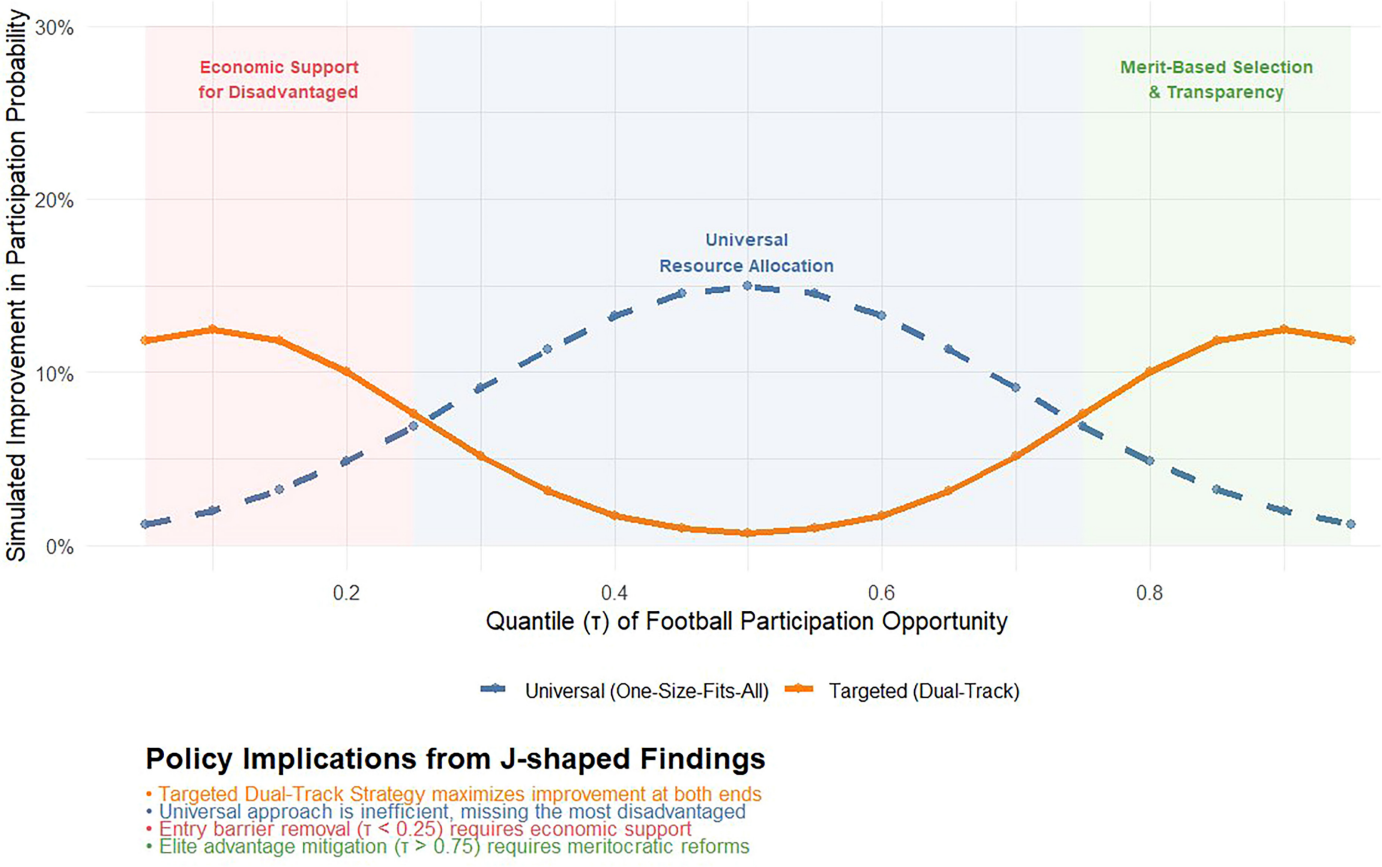
Simulated policy effects: universal vs. targeted dual-track strategies. This simulation compares the effectiveness of two policy approaches in improving football participation probability across the opportunity distribution. The dashed blue line represents a “Universal” (one-size-fits-all) resource allocation strategy, showing moderate improvement in the middle of the distribution but minimal impact at both tails. The solid orange line represents a “targeted dual-track” strategy informed by the J-shaped findings, combining economic support for disadvantaged adolescents (τ < 0.25) with meritocratic reforms for elite selection (τ < 0.75). This targeted approach yields superior overall improvement, particularly addressing inequality at both ends.

Finally, to translate the observed empirical patterns into potential policy insights, we created a policy implication simulation map (Figure 6). The figure compares the conceptual effectiveness of a “universal” resource allocation policy with a “targeted two-track” policy inspired by our J-shaped results. Simulated curves are generated by applying hypothetical effect sizes to a normalized quantile distribution, with effectiveness expressed in terms of probability of participation chance improvement for schematic purposes only.

For comparison, we also fit a standard ordinary least squares regression model. To address potential concerns regarding the construction of the Football Participation Opportunity Index, we re-ran our primary model (OLS model and quantile regression with fixed effects at τ = 0.10, 0.50, 0.90) using two alternative outcome measures. Sensitivity analyses were conducted (a) using a linear probability model with fixed effects to analyze the binary measure of “past involvement in organized football” and (B) on the continuous “depth” measure of participant-only analysis (hours per week). The J-shaped pattern of economic capital coefficients remains broadly consistent under these alternative settings, thereby reinforcing confidence in our main findings. All statistical calculations were done using the qreg2 command of the Stata software (version 18.0). All graphical visualizations were made using R software (version 4.4.2) with the packages ggplot2 (v3.5.0), ggcorrplot (v0.1.4), cowplot (v1.1.1), and patchwork (v1.2.0).

### 2.5 Robustness check

To assess the robustness of our main findings, we performed two complementary analyses. First, we estimated an ordinary least squares (OLS) model with an interaction term between the Economic Capital Index (ECI) and a binary indicator of high soccer chance (median split based on FOI) to test whether the differential effect can be captured in parametric form. Second, we re-estimated our core quantile regression model using proxies for key variables: (a) using only parental education level as a simpler proxy for socioeconomic status, and (B) decomposing the composite FOI, analyzing its “depth” component (hours per week) as a continuous outcome.

## 3 Results

### 3.1 Sample characteristics and univariate analysis

Table 1 presents the characteristics of study participants stratified by the Football Participation Opportunity Index tertiles. Univariate analysis revealed statistically significant gradient differences for almost all of the measured variables, clearly delineating the contours of systemic inequality. To further illustrate the distribution patterns of the key variables, Figure 1 demonstrates the distribution of the Football Participation Opportunity Index and the Household Economic Capital Index and their correlations with other covariates. The histograms in Figures 1A,B confirm that both the Football Participation Opportunity Index and the Household Economic Capital Index are approximately normally distributed in our sample. The correlation matrix (Figure 1C) shows that higher football participation opportunity index is positively related to urban residence, parental education and adequate school facilities, while it is negatively related to academic pressure, which provides a bivariate perspective for our preliminary understanding of the ecosystem of unequal sports opportunities.

**TABLE 1:** Characteristics of the study participants by tertiles of football participation opportunity index (FOI) (*n* = 1,860).

Characteristic	Total sample	Low FOI tertile (*n* = 620)	Medium FOI tertile (*n* = 620)	High FOI tertile (*n* = 620)	*F*/χ ^2^	*p*-value
Individual factors						
Age, mean (SD)	15.4 (1.7)	15.6 (1.8)	15.3 (1.7)	15.2 (1.6)	*F* = 12.47	< 0.001
Male, %	52.5	40.5	52.9	64.2	χ^2^ = 78.32	< 0.001
Urban residence, %	58.9	39.8	58.5	78.4	χ^2^ = 185.64	< 0.001
Only child, %	63.8	53.7	64.5	73.1	χ^2^ = 52.18	< 0.001
Self-rated health (good/very good), %	68.5	55.2	70.3	80.0	χ^2^ = 92.15	< 0.001
BMI category (overweight/obese), %	21.3	25.6	20.2	18.1	χ^2^ = 11.52	0.003
Family capital factors						
Economic capital index, mean (SD)	0.02 (0.96)	−0.75 (0.85)	0.03 (0.92)	0.78 (0.80)	*F* = 498.26	< 0.001
Parental education (college +),%	41.5	25.8	42.1	56.6	χ^2^ = 136.90	< 0.001
Parental sports participation, %	32.2	16.3	32.4	48.2	χ^2^ = 166.34	< 0.001
Family sports equipment, mean (SD)	2.5 (1.3)	1.8 (1.1)	2.6 (1.2)	3.2 (1.2)	*F* = 243.18	< 0.001
Parental social network (in sports), %	18.9	8.1	18.7	30.0	χ^2^ = 112.58	< 0.001
School and environmental factors						
School sports facility (adequate), %	59.3	32.9	60.2	85.1	χ^2^ = 329.71	< 0.001
Peer support for PA, mean (SD)	3.6 (1.0)	3.0 (1.1)	3.7 (0.9)	4.2 (0.8)	*F* = 295.33	< 0.001
Weekly academic stress, mean (SD)	3.9 (1.1)	4.2 (1.0)	3.9 (1.1)	3.7 (1.1)	*F* = 25.89	< 0.001
Community sports center access, %	55.1	42.1	55.6	67.6	χ^2^ = 88.27	< 0.001

### 3.2 Conceptual justification for the analytical approach

Before presenting the core multivariate results, Figure 2 provides a conceptual visualization to justify the use of quantile regression with school-level fixed effects. Figure 2A demonstrates the traditional pooled ordinary least squares regression analysis, which estimates the single mean association of economic capital with football participation across all schools. This approach masks the significant inter-school heterogeneity visible in the scatter plot. By contrast, Figure 2B illustrates the principle of our chosen approach: after applying school-level fixed effects to remove school-specific factors that do not change over time, the relationship between economic capital and football participation within schools is non-linear and heterogeneous. This visualization highlights the inadequacy of the standard ordinary least squares model to capture the complex, context-dependent relationships we hypothesized, necessitating the adoption of a quantile regression framework.

### 3.3 Multivariable analysis: the j-shaped heterogeneous effect of economic capital

The core results of the multivariate analysis are summarized in Table 2. After adjusting for all covariates and school-level fixed effects, the ordinary least squares model indicated a significant positive correlation between the Economic Capital Index and the Football Participation Opportunity Index (β = 0.192, *p* < 0.001). However, a quantile regression model with school fixed effects revealed a more nuanced and statistically significant heterogeneous relationship (Wald test for equality across quantile coefficients: χ^2^ = 31.7, *p* < 0.001). The nature of this heterogeneity is strongly illustrated in Figure 3, which plots the variation of the economic capital coefficient across the conditional distribution of football chances (quantile τ from 0.05 to 0.95). The coefficient exhibits a clear J-shaped curve: it is largest at the 10th quantile (τ = 0.10; β = 0.341, 95% CI: 0.250–0.432), weakened around the median (τ = 0.50; β = 0.170, 95% CI: 0.095–0.245) and again enhanced at the 90th quantile (τ = 0.90; β = 0.260, 95% CI: 0.145–0.375). The embedded illustration in Figure 3 directly contrasts the single slope estimated by ordinary least squares (representing the “mean association”) with the multiple changing slopes captured by quantile regression at different points of the football participation chance index distribution, visually reinforcing the methodological advantages of our method.

**TABLE 2:** Multivariable analysis: OLS and quantile regression for economic capital index.

Model	β−coefficient	95% CI	*p*-value
OLS (average effect)	0.192	(0.137, 0.247)	< 0.001
Quantile regression			
τ = 0.10	0.341	(0.250, 0.432)	< 0.001
τ = 0.25	0.245	(0.173, 0.317)	< 0.001
τ = 0.50	0.170	(0.095, 0.245)	< 0.001
τ = 0.75	0.201	(0.121, 0.281)	< 0.001
τ = 0.90	0.260	(0.145, 0.384)	< 0.001

All models are adjusted for the full set of covariates listed in Table 1 and include school-level fixed effects. OLS, ordinary least squares.

Robustness checks supported the main findings. The interaction term in the OLS model was positive and significant (β = 0.121, *p* = 0.008), confirming a stronger association between economic capital and participation at higher opportunity levels. Furthermore, J-shaped coefficient patterns remained qualitatively consistent when using parental education as a core predictor and when analyzing ongoing “depth” of engagement, further confirming that the observed heterogeneity was not an artifact of our specific composite index construction.

### 3.4 Mechanistic pathways underlying the j-shaped pattern

To resolve the dual role of economic capital implied by the J-curve, Figure 4 illustrates the hypothetical mechanism path. This diagram conceptualizes how household economic capital relates to football participation opportunities through two different channels. For adolescents at the bottom of the opportunity distribution (left path), economic deprivation may create “barriers to access” in the form of direct cost barriers (equipment, fees), high opportunity costs perceived in a culture that emphasizes academic stress, and a sense of social exclusion. For adolescents at the top of the distribution (the right path), economic surplus may contribute to the “elite promotion ladder” effect, in which resources are strategically transformed into personal coaching, access to elite competition platforms and valuable social capital in sports networks. This figure goes beyond statistical association and proposes a conceptual causal framework for explaining quantile regression results, linking economic resource differences to specific social and psychological exclusion and advantage mechanisms, which is worthy of future research.

### 3.5 Heterogeneous effects of contextual covariates across the opportunity spectrum

Table 3 presents the estimated coefficients for all covariates at selected quantiles (τ = 0.10, 0.50, 0.90) from the quantile regression model with school fixed effects. Extending this analysis, Figure 5 visually traces the trajectory of coefficients for four key covariates—urban residence, academic stress, parental social network, and school facilities—across the opportunity spectrum (τ = 0.05–0.95), revealing different types of associations within the PE inequality ecosystem. The analysis identified “access-sensitive” factors (urban residence and academic stress), which are most strongly associated at the lower end of the distribution (τ≈ 0.10) and therefore may constitute a major barrier to initial participation. In contrast, the “elite-sensitive” factor (parental social network) is significantly enhanced at the high end of the distribution (τ > 0.75) and may act as a catalyst for access to advanced opportunities. In contrast, the “universal facilitative” factor (adequate school facilities) showed a consistent and significant positive correlation across all quantiles, highlighting the fundamental importance of these factors for participation regardless of the adolescent’s starting point. The visualization provided by Figure 5 complements the discrete point estimates in Table 3 by clarifying the continuous trend of these associations across the conditional distribution. This graphical representation allows for a more nuanced and dynamic categorization of how various contextual factors differentially influence patterns of football participation opportunities, going beyond static comparisons on isolated quantiles to reveal how the nature and strength of each factor’s associations evolve across the spectrum of strengths and weaknesses.

**TABLE 3:** Quantile regression analysis of covariate effects on football participation opportunity at selected quantiles.

Covariate	τ = 0.10 (95% CI)	τ = 0.50 (95% CI)	τ = 0.90 (95% CI)
Individual factors			
Age	−0.035 (−0.078, 0.008)	−0.015 (−0.045, 0.015)	−0.010 (−0.065, 0.045)
Male	0.388 (0.285, 0.491)*	0.421 (0.341, 0.501)*	0.451 (0.320, 0.582)*
Urban residence	0.251 (0.142, 0.360)*	0.165 (0.080, 0.250)*	0.128 (−0.020, 0.276)
Only child	0.045 (−0.052, 0.142)	0.041 (−0.035, 0.117)	0.025 (−0.100, 0.150)
Good self-rated health	0.128 (0.045, 0.211)*	0.098 (0.030, 0.166)*	0.085 (−0.030, 0.200)
Overweight/obese	−0.078 (−0.185, 0.029)	−0.055 (−0.135, 0.025)	−0.041 (−0.175, 0.093)
Family capital factors			
Parental education (College +)	0.075 (−0.030, 0.180)	0.095 (0.010, 0.180)*	0.110 (0.005, 0.215)*
Parental sports participation	0.185 (0.090, 0.280)*	0.220 (0.145, 0.295)*	0.235 (0.120, 0.350)*
Family sports equipment	0.065 (0.005, 0.125)*	0.052 (0.005, 0.099)*	0.045 (−0.025, 0.115)
Parental social network	0.095 (−0.010, 0.200)	0.135 (0.050, 0.220)*	0.155 (0.030, 0.280)*
School and environmental factors			
School facility (Adequate)	0.315 (0.215, 0.415)*	0.275 (0.195, 0.355)*	0.245 (0.125, 0.365)*
Peer support for PA	0.105 (0.045, 0.165)*	0.118 (0.070, 0.166)*	0.125 (0.055, 0.195)*
Weekly academic stress	−0.085(−0.145, −0.025)*	−0.072 (−0.120, −0.024)*	−0.060 (−0.130, 0.010)
Community center access	0.125 (0.035, 0.215)*	0.088 (0.010, 0.166)*	0.065 (−0.045, 0.175)

All models include school-level fixed effects. * Indicates *p* < 0.05. CI, confidence interval. PA, physical activity.

### 3.6 Policy implications derived from the heterogeneous effects

To translate the observed empirical patterns into potentially viable insights, Figure 6 illustrates the conceptual impact of two different policy approaches in improving the probability of football participation across the opportunity distribution. The dotted line represents the “universal” (one-size-fits-all) resource allocation strategy, which shows a moderate effect in the middle of the distribution but fails to address the urgent needs at both ends. The solid line represents the “targeted dual-track” strategy inspired by our J-shaped research findings. The approach combines economic support targeted at the most disadvantaged (τ < 0.25) to remove barriers to access, with institutional reforms aimed at ensuring meritocracy and transparency in the elite selection process for the most advantaged (τ > 0.75). The simulation results show that, conceptually, targeting strategies can lead to better overall improvement, especially by addressing the most acute nodes of inequality, thus providing a visual basis for considering precise public health interventions in youth sports.

## 4 Discussion

Using quantile regression combined with school-level fixed effects, supplemented by a series of tailor-made visual charts, this study reveals a significant and complex relationship between household economic capital and youth football participation opportunities in Jilin Province, China. Our central finding, namely a distinct J-type pattern of heterogeneous associations, not only responds to the main research question, but also greatly deepens the understanding based on traditional mean analysis. The visual narrative constructed through Figures 1–6 goes beyond the tabulated results to provide an intuitive and powerful discussion of the shape of inequality, its proposed mechanisms, and its potential policy implications.

### 4.1 Key findings and visual interpretation in context

The central finding of this study is that the coefficient of economic capital is largest at both ends of the opportunity distribution, but there is a fundamental asymmetry in its nature and strength. The coefficient at the bottom end (τ = 0.10; β = 0.341) is significantly larger than that at the top end (τ = 0.90; β = 0.260). This J-type pattern, as vividly depicted in Figure 3, is in sharp contrast to the conclusion implied by the simple linear fit of the hybrid OLS model shown in the inset of the figure. Our visual analysis shows that the average positive association is an integrated result, which may be driven by strong associations at both ends of the distribution. This key insight is clearly visible in the elevated left tail of the curve, suggesting that a blanket allocation of resources to “youth football” may be inefficient because it masks the specific subgroup facing the most robust socioeconomic barriers: those youth with extremely low opportunities.

This finding resonates strongly with the root cause theory. According to this theory, resources such as money become the root cause of health inequality when they can flexibly obtain benefits in different situations (Øversveen et al., 2017). Our study provides a visual framework consistent with this flexibility through the conceptual mapping in Figure 4: economic capital is associated with outcomes at both ends of the opportunity spectrum. At the bottom, its presence (or scarcity) is tied to the basic preconditions for participation; at the top, its abundance is tied to competitive advantage. As observed, this pattern of association indicates the ability to obtain context-specific benefits in different social strata, as illustrated by the different paths in the schematic diagram, which is the sign of the root cause (Clouston et al., 2016).

Our findings, especially the J-pattern visualized in Figure 3 and the dual-path framework conceptualized in Figure 4, provide a novel empirical illustration of the “resource flexibility” principle of root cause theory. We demonstrate that economic capital is not only associated with better average outcomes, but that its association exhibits the greatest strength at both ends of the chance distribution. This pattern suggests that the same resource is deployed flexibly in fundamentally different ways—overcoming absolute deprivation at the bottom and ensuring relative advantage at the top—a subtle difference in distribution that is often masked by mean-based analysis (Phelan et al., 2010). Thus, our study extends the theory by mapping how an underlying cause can act as both a low-end “barrier” and a high-end “ladder” on a continuum of health-related opportunities. Based on this theoretical understanding, the next section will provide an in-depth analysis of the mechanisms that may constitute this J-shaped pattern.

### 4.2 In-depth mechanistic analysis of the j-shaped pattern

The large coefficient of economic capital at the lowest quantile—highlighted in Figure 3 and path-mapped in Figure 4—is consistent with its potential role as a large “barrier to entry.” This barrier may operate through multiple, interrelated mechanisms that go beyond simple direct costs. While the inability to afford equipment and expenses constitutes the most immediate barrier (Dennaoui et al., 2024), the barrier is likely to be reinforced by significant psychological and social forces. For families with limited resources, the opportunity cost of investing in sport is considered exceptionally high in a culture that places a high value on academic achievement (Su et al., 2022). This trade-off may be further exacerbated by the cognitive load imposed by scarcity, which consumes the mental bandwidth needed for parents to cope with complex extracurricular inputs (Ren et al., 2023). At the same time, adolescents may experience social exclusion or shame, which leads to self-withdrawal from activities (Spruijtenburg et al., 2024). Thus, the barriers suggested by our data are not merely economic, but point to a complex interplay between economic constraints, cognitive scarcities, and symbolic boundaries.

At the other end of the spectrum, for adolescents who already have a large number of opportunities to participate, economic capital is associated with outcomes that fit the “elite ladder” and may push them toward more selective levels of competition. Figure 4 conceptualizes this potential shift as a shift from ensuring basic access to cultivating competitive advantage. Families may engage in collaborative parenting, strategically allocating resources to ensure access to personal coaching and high-level platforms (Ilari et al., 2022). This investment may promote capital transformation, that is, the transformation of economic capital into internalized cultural capital (such as exquisite skills) and into valuable social capital through influential relationships (Kärtner and von Suchodoletz, 2021). These transformed forms of capital can then play a role in selective institutional systems (Luo et al., 2025). Therefore, at this level, the association of economic capital may play a role in magnifying the existing advantages, which is a model consistent with the reproduction of social opportunities.

### 4.3 The heterogeneous roles of contextual covariates and their visual typology

The complex association of economic capital is further contextualized by the heterogeneous association of other covariates, which together paint a complete picture of the sports inequality ecosystem. Figure 5 pushes this understanding beyond discrete quantile comparisons by visually tracing continuous coefficient trajectories, allowing us to classify covariates into different types. The rural-urban divide appears primarily as an “access-sensitive” barrier; the strong association of urban residence at the lowest quantile (clearly visible in Figure 5) highlights how infrastructure differences are closely linked to the systemic exclusion of rural adolescents (Tian et al., 2021). In contrast, parental social networks function as “elite-sensitive” promotion ladders; their increased relevance at higher quantiles points to the role of Guanxi in opening selective channels (Barbalet, 2025). At the same time, the negative association of academic stress in the lowest quantile suggests that China’s high-risk education system is most strongly associated with the phenomenon of the most vulnerable youth being squeezed out of sports participation (Zhu et al., 2021). Corresponding to these barriers to change, factors such as adequate school facilities play a “universal promotion” role and show sustained importance throughout the spectrum (Eime et al., 2015). The system of these complementary and mutually reinforcing factors, after visual classification, depicts a pattern: household economic capital shows the strongest correlation at the two key nodes of participation access and elite selection.

### 4.4 Implications for public health policy and practice: from visualization to simulation

Our findings provide timely insights into the national sports policy framework in China, especially the “Football Reform and Development” program and the “School Football” initiative. Observed J-shaped associations suggest that achieving the dual goals of these policies–broad participation and elite creation—requires confronting the dual role of economic capital. Strong “barriers to entry” at the low end of the distribution suggest that universal promotion may not adequately cover the most disadvantaged youth, which may compromise equity goals. At the same time, the “elite ladder” effect at the high end suggests that the path to excellence may be skewed by economic advantages, which could affect the development of talent at the core of China’s long-term football aspirations.

Our analysis of J-ties provides unprecedented detail for public health interventions, supporting a shift from one-size-fits-all policies to more precise strategies. Figure 6 directly translates this analytical insight into a tool for policy consideration, suggesting a complex two-pronged strategy. Such an approach may need to simultaneously address the economic “barriers to entry” faced by the most vulnerable and weaken the inequitable advantages associated with the “meritocratic ladder.” To effectively lower the participation threshold, public health policies can prioritize strong economic interventions, including targeted subsidies for low-income households (Kohl et al., 2013), and guarantee access to free, high-quality equipment in schools (Farooq et al., 2018). At the same time, to address the distortions implied by the top, the policy focus could shift to institutional reform to ensure fair and merit-based selection of the elite. This includes regulating private training institutions while establishing needs-based scholarships (Pfeiffer and Clevenger, 2024), implementing a “blind selection” process (Till and Baker, 2020), and strengthening public investment in grassroots facilities by the government (Whitley et al., 2019). As shown conceptually in Figure 6, this targeted dual-track strategy is expected to bring better overall results than universal approach, graphically demonstrating the value of precise targeting based on heterogeneous effect analysis in the field of public health.

### 4.5 Limitations and strengths

Some limitations of this study should be recognized. First, the cross-sectional design precludes exact causal inference; observed associations should be interpreted as descriptions of patterns rather than causal effects. Second, despite controlling for rich covariates and employing school fixed effects, there may be residual confounding, particularly from unobserved family-level factors (e.g., parental attitudes). Third, although validated, the construction of the integrated FOI may mask the specific contributions of the individual components; nevertheless, our sensitivity analysis supports the robustness of the core model. Fourth, the study focuses on Jilin Province, so it needs to be cautious when it is extended to other regions with different socio-economic and sports policy backgrounds in China. Fifth, the use of quantile regression on multiple quantiles and covariates is an exploratory analysis and is not corrected for multiple testing. It is worth noting that this study has significant advantages in terms of methodology and dissemination. By combining quantile regression incorporating school-level fixed effects with strategic visual narratives (Figures 1–6), we not only identify a complex pattern of inequality, but make it comprehensible and actionable to a multidisciplinary audience. These visualizations help clarify methodological principles (Figure 2), present core findings clearly (Figure 3), propose testable mechanistic frameworks (Figure 4), categorize contextual influences (Figure 5), and bridge research and policy considerations (Figure 6). This approach enhances the translational potential of our research findings in public health and health equity.

## 5 Conclusion

Through methodological innovation and integrated visual storytelling, this study demonstrates that the association between family economic capital and youth football participation follows a profound J-shaped pattern of heterogeneity. This model is consistent with the view that economic capital can be used as both an exclusive “access barrier” and a differentiated “elite promotion ladder” associated with the outcome. This finding goes beyond the limitations of traditional mean-based analysis and provides a fine map of inequality. It suggests that public health strategies to promote equity in sport should consider a two-track approach: one that applies strong economic support to address absolute deprivation at the bottom, and another that engages in structural reform to address relative advantage at the top. Only through this targeted and parallel approach can we effectively respond to the multi-level and dynamic nature of inequality in youth sports and truly advance the goal of health equity. The visual narrative framework enhances the explanatory power and policy relevance of complex empirical findings, and provides a methodological reference for similar studies.

## Data Availability

The raw data supporting the conclusions of this article will be made available by the authors, without undue reservation.
